# Smartphones and Apps to Control Glycosylated Hemoglobin (HbA1c) Level in Diabetes: A Systematic Review and Meta-Analysis

**DOI:** 10.3390/jcm9030693

**Published:** 2020-03-04

**Authors:** María Begoña Martos-Cabrera, Almudena Velando-Soriano, Laura Pradas-Hernández, Nora Suleiman-Martos, Guillermo A. Cañadas-De la Fuente, Luis Albendín-García, José L. Gómez-Urquiza

**Affiliations:** 1 San Cecilio Clinical University Hospital, Andalusian Health Service, Avenida del Conocimiento, s/n, 18016 Granada, Spain; bego_martos@hotmail.com; 2 Benamaurel, Northeast District of Granada, Andalusian Health Service, 18817 Granada, Spain; srtavelando@gmail.com; 3 Las Gabias Health Center, Granada Metropolitan District, Andalusian-Health Service, Plaza Montes Jovellar S.N, 18110. Granada, Spain; lauraphl9@gmail.com; 4 Faculty of Health Sciences, University of Granada, Calle Cortadura del Valle S.N., 51001 Ceuta, Spain; norasm@ugr.es; 5 Faculty of Health Sciences, University of Granada, Avenida de la Ilustración, 60, 18016 Granada, Spain; gacf@ugr.es (G.A.C.-D.l.F.); jlgurquiza@ugr.es (J.L.G.-U.); 6 La Chana Health Center, Granada Metropolitan District, Andalusian Health Service, Calle Virgen de la Consolación, 12, 18015 Granada, Spain

**Keywords:** glycosylated hemoglobin, health education, meta-analysis, prevention and control, diabetes mellitus, smartphone application

## Abstract

*Introduction:* Diabetes mellitus is a chronic endocrine-metabolic disease, the evolution of which is closely related to people’s self-control of glycemic levels through nutrition, exercise, and medicines. *Aim:* To determine whether smartphone apps can help persons with diabetes to improve their % levels of glycosylated hemoglobin. *Method:* A systematic review and meta-analysis were done. ProQuest, Pubmed/Medline, and Scopus databases were used. The search equation used was “(Prevention and Control) AND Diabetes Mellitus AND Smartphones”. The inclusion criteria applied were clinical trials, conducted in 2014–2019. *Results: n* = 18 studies were included in the review. The studies tried different applications to monitor glycemia and support patients to improve glycosylated hemoglobin (HbA1c) levels. More than half of the studies found statistically significant differences in HbA1c in the intervention group compared with the control group. Eleven studies were included in the meta-analysis and the study sample was *n* = 545 for the experimental group and *n* = 454 for the control group. The meta-analytic estimation of the HbA1c % level means differences between intervention and control group was statistically significant in favour of the intervention group with a mean difference of –0.37 (–0.58, –0.15. 95% confidence interval). *Conclusion:* Smartphone apps can help people with diabetes to improve their level of HbA1c, but the clinical impact is low.

## 1. Introduction

Diabetes mellitus (DM) is a chronic endocrine-metabolic disease that is characterised by poor insulin secretion or by the inability of the body’s cells to respond to it. This condition results in high blood sugar levels, which in the long term can trigger disabling and dangerous medical complications. There are three main types of diabetes: type 1, type 2, and gestational. Type 1 (DM1) is caused by an autoimmune reaction that attacks the pancreatic beta cells, which are responsible for the production of insulin. DM1 cannot be prevented or cured, but action can be taken to avoid complications and premature death. In type 2 diabetes (DM2), the cells of the target organs develop resistance to insulin; moreover, its production by the pancreatic beta cells is often insufficient. DM2 accounts for about 90% of all cases of DM. However, certain risk factors, such as obesity, the consumption of sugary drinks, and the lack of physical activity, can be addressed to modify, delay, or prevent its appearance [1,2,3,4].

DM is one of the most prevalent diseases worldwide, affecting 425 million people. Also, an estimated 212.4 million cases remain undiagnosed [3]. According to various studies, the prevalence of diabetes and the resulting levels of mortality and healthcare expenditure will continue to increase, producing a major social, medical, and economic impact. The total DM-related health expenditure in 2015 was an estimated 673 billion US dollars, and this figure is expected to reach 802 billion by 2040. DM causes up to five million deaths a year, worldwide. It is in middle- to low-income countries, i.e., those with fewest resources, where most DM-related deaths among the younger population are recorded and where its prevalence is increasing fastest. The World Health Organization estimates that by 2025 this prevalence will have increased by 42% in developed countries and by 170% in developing countries [1,2,5].

To prevent DM-related complications and deaths, healthcare authorities should promote the adoption of healthy lifestyles. In addition, the detection, early treatment, and proper control of the disease should be encouraged. To complement these advances, adequate health care must be provided, including the necessary resources to improve quantity and quality of life [6].

In this respect, smartphone applications (apps), understood in this study as a program that can be installed in a smartphone and that has been designed for health care in people with diabetes, may play a very useful role, contributing to DM2 prevention and preventing the adverse effects of DM1 and DM2, increasing the quality of care provided to people with diabetes and their families, facilitating the acquisition of knowledge, increasing treatment adherence, and helping patients avoid complications. The use of such apps would improve patients’ quality of life by slowing the onset of the DM2 and/or both DM complications. However, users must be well informed of the different apps available to determine which one is most appropriate for their situation [1,7,8]. App developers also need to take into account that these advances should be flexible in addressing the personality and specific problems of each user so they can afford patients different solutions to improve their diabetes self-management [9].

This study has the following main aim: to determine whether the use of an appropriate smartphone application can help people with diabetes to improve their level of glycosylated hemoglobin (HbA1c).

## 2. Material and Methods

### 2.1. Design

A systematic review with meta-analysis was done following the PRISMA recommendations [10].

#### 2.1.1. Search Strategy

A bibliographic search was performed in ProQuest, Medline, and Scopus databases. The search terms used were “(Prevention and control) AND Diabetes Mellitus AND Smartphones”. The descriptors in the search equation were obtained from the Medical Subject Headings (MeSH) thesaurus.

#### 2.1.2. Study Selection

The following inclusion criteria were applied: full-text clinical trials, focusing on how smartphone apps or educational or monitoring programs can be used by patients with DM to control/improve their level of glycosylated hemoglobin (HbA1c); published in English or Spanish, during the period from January 2014 to December 2019.

Studies focusing on the prevention of other chronic non-contagious diseases (such as obesity, high blood pressure, or hypercholesterolaemia) and those describing clinical trials that are ongoing and have not yet reported results were excluded.

The study search and selection processes were carried out by two members of the research team, working independently; any disagreements were resolved by consulting a third member. After the initial extraction of studies by the data search, in every case, the title and abstract were read. The papers considered potentially suitable for inclusion were then read in their entirety. To find more potential studies to include in the review, an inverse (searching in the references of the studies) and a forward search were done with the included studies. An inverse search was also done in the references of all the systematic reviews about diabetes that were found in the search.

#### 2.1.3. Data Extraction

To extract the data of each included study, a data worksheet was created including the following information: the first author, year of publication, country of the study, study type, sample, intervention, main results.

The main outcome of the review and the meta-analysis was the % of HbA1c in people with diabetes.

### 2.2. Critical Review and Level of Evidence

The quality of the studies included in this review was assessed following the levels of evidence and degrees of recommendation stipulated by the Oxford Centre for Evidence-Based Medicine (OCEBM) [11].

### 2.3. Data Analysis

A descriptive analysis of the studies included in the systematic review was done. For the meta-analysis, Review Manager 5.3 software [12] was used. A random-effects meta-analysis was done. Egger test bias was used for assessing publication bias and the I2 test was used for assessing heterogeneity level. 

## 3. Results

**Figure 1:**
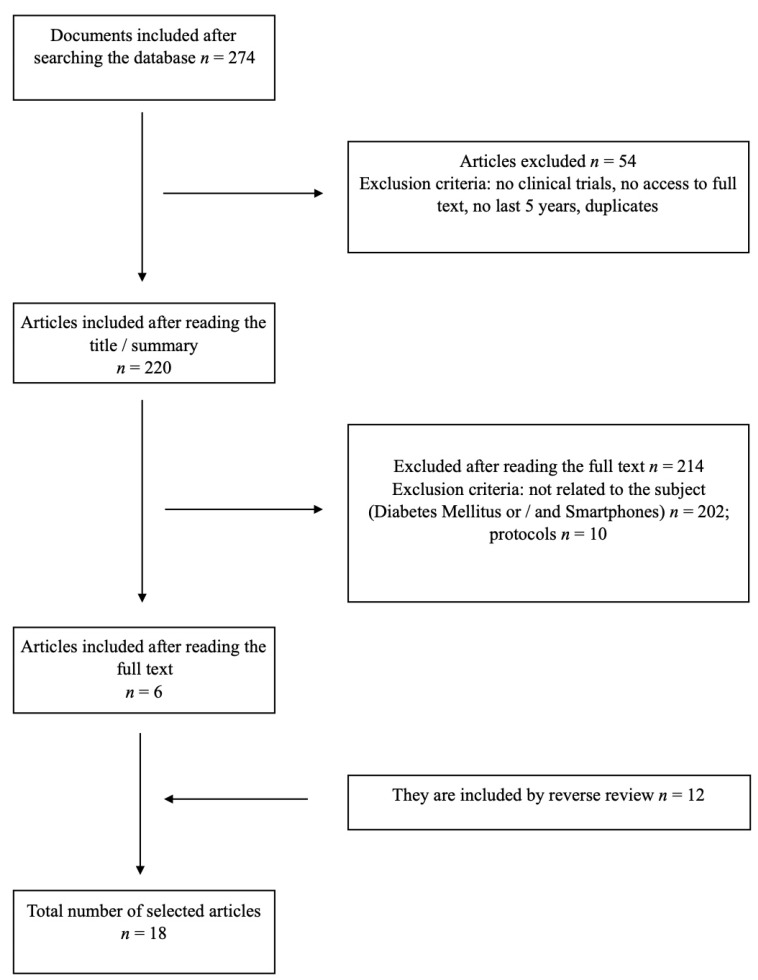
Flowchart of the study selection process.

The database search obtained 278 articles, of which 54 were duplicates, were not clinical trials, or lacked full-text access. These 54, therefore, were excluded. After reading the titles and abstracts, a further 214 were excluded because they did not meet the inclusion criteria or referred to clinical trials that had not yet obtained results. Thus, six papers remained. A reverse and forward search was then conducted, which revealed another twelve valid articles. Thus, the final sample of studies for the systematic review was *n* = 18 and *n* = 11 for the meta-analysis (File S1). The flow chart of the study selection process is shown in Figure 1.

All of the studies were random clinical trials. Ten studies focused on people with DM2, another five in people with DM1, two on people with DM1 and DM2, and one in women with gestational diabetes. The observation time elapsed from the start of the intervention to the measurement of the results obtained ranged from 14 days to 12 months. Blood sugar was monitored to reveal post-intervention objective changes in glycosylated hemoglobin (HbA1c) and is expressed in this review and the meta-analysis as a %, not in mmol/mol. All of the included studies are detailed in Table 1.

### 3.1. Apps and Mobile Phones for the Control of DM1

One study [13] used the VoiceDiab^®^ app, which helps users calculate the bolus insulin needed to avoid hypo and hyperglycemia by entering blood glucose data (which can be voice-entered). The app tries to help patients to increase the glycemic time-in-range, within the recommended 70–180 mg/dL. However, after the intervention, no significant changes were found in the HbA1c levels between the control group (6.98) and the intervention group (7.10) [13]. In the study with children and adolescents done by Klee et al., they found that the patients that used the app Webdia (designed for metabolic control in diabetes 1) decreased their level of HbA1c by 0.33 (±0.75), while the HbA1c level increased with the usual care 0.21 (±0.79) [14].

Other support systems, such as connection to an internet platform, require a greater commitment by the patient. In one study in this respect, the patients supplied health data, namely current blood sugar levels and the diet consumed, and the internet platform provided feedback on questions, such as the insulin to be administered or the amount of physical exercise that could be performed. Although this support was not significantly related to improved blood sugar levels, it did greatly facilitate the patients’ monitoring of their status. This provision of additional information on health status, therefore, helps correct glycemic instability [15]. Similar results were found by Drion et al. [16] using the Diabetes Under Control app for 3 months, because at the end of the intervention the differences between the intervention and the control groups were not significant. Similar negative results were shown when comparing both groups at the end of the intervention in the study of Skrovset al. using Diabetes Diary App for 18 weeks [17]. 

### 3.2. Apps and Mobile Phones for the Control of DM2 

One study [18] with patients with diabetes mellitus 2 used “The Few Touch App” as a diabetes diary which was designed to increase self-management through awareness, an overview of relevant factors, and motivational feedback. With the use of this application for one year, the HbA1c level decreased by 0.31. This decrease in the HbA1c level was higher than the decrease in the control group (0.16) and the other intervention group, which used the app and health counseling (0.15). However, the differences between groups did not significantly differ [18]. Differing results were found in a 24 weeks clinical trial with the app mDiabetes [19], which used an algorithm to send immediate feedback messages according to the glycemic control that the patients have sent. The control group (which used a manual book for recording their glycemic levels) showed a lower reduction after the 24 weeks in the HbA1c level of 0.06 (±0.1), while the intervention group showed a 0.40 (±0.09) reduction. The differences between groups were 0.35 with *p* < 0.05. Another smartphone application, Dnurse App, used with pregnant women with gestational diabetes to monitor fasting and post-prandial glucose, and including online instruction (done by a nurse every day for 2 hours to answer questions about diet, exercise, blood glucose, and other relevant topics) and notifications if they uploaded an abnormal blood glucose result, showed positive results in HbA1c levels [20]. The level in the control group was higher than the intervention group with the app (5.3 vs. 4.7) with *p* < 0.05 [20].

Some patients diagnosed with DM2 use not only a smartphone app but also telemedicine (i.e., direct communication) to receive educational interventions in self-management of the disease, coaching, and to practice skills related to diet and exercise. Karhula et al. [21] used a mobile application for telemonitorization and phone-based coaching to improve diabetes management. The difference between the pre- and post-intervention HbA1c level was an increase of 0.04 in the intervention group and 0.18 in the control group without statistically significant differences between both groups at the end of the study (*p* > 0.05) [21]. Different results were found by one study of patients who received regular communication via text messages and phone calls, and therefore it was concluded that this assistance was beneficial, leading to reduced levels of HbA1c (pre-intervention, 6.96 ± 1.3; post-intervention, 6.55 ± 1.06) [22].

In another study of the use of mobile phones to help patients with diabetes control their blood sugar levels, the data provided (on blood sugar, weight, and blood pressure) enabled them to receive practical recommendations, supported by text messages or phone calls to remind the patient when and how this advice should be applied. The use of this platform led to decreased levels of HbA1c (pre-intervention, 8.44 ± 1.58; three months post-intervention, 6.84 ± 1.2) [23]. Another study that used a cloud-based diabetes management app for 14 weeks showed positive results decreased HbA1c and, the differences with the control group at the end of the intervention were statistically significant [24]. 

Another app that is used similarly is “DialBetics^®^”. In this case, data for glycemia, blood pressure, body weight, and steps (counted by a pedometer) were analyzed to create personalized objectives. Patients then received messages with dietary and exercise advice. This intervention also improved HbA1c results, with a decrease of 0.4 points (pre-intervention, 7.1 ± 1; three months post-intervention, 6.7 ± 0.7) [25]. Another study that used the mobile phone to send messages to enhance patient motivation, self-efficacy, and ability to perform diabetes self-care behaviors showed no statistically significant effect when comparing the results with the control group after the 6 month intervention [26]. Results also showing no statistical significance were shown in a study in Norway (using the Few Touch Application for 4 months as a diary for type 2 diabetes included in a smartphone) [27] and in one study in Canada where patients recorded their information about blood glucose, physical activity, food intake, and mood in an app and were also in touch with their healthcare professionals [28]. In Mexico, another project used the “Brew app”, which was connected with patients´ glucose meter information and allowed them to take interactive surveys, read text messages, watch educational videos, or read brochures [29]. After 10 months the intervention group reduced their HbA1c mean by –3.02(± 2.83), while the decrease in the control group was –1.30(± 0.3.29); the differences between both groups were statistically significant [29]. Similarly, Zhou et al. [30] found significant differences in the HbA1c levels between the intervention and the control group after using the Welltang app for 3 months (a diabetes management application that can be used by patients and clinicians for knowledge, self-management, and communication between patients and clinicians).

### 3.3. Meta-Analysis Results

Eleven studies were included in the meta-analysis. The study sample was *n* = 545 for the experimental group and *n* = 454 for the control group. The included studies used telemedicine through apps, messages, and calls for supporting people with diabetes. Seven studies [14,16,17,18,22,26,29] included in the systematic review were not included in the meta-analysis because they did not report all the necessary data.

An Egger test did not show publication bias (*p* > 0.05). The heterogeneity analysis was high *I*^2^ = 73%. 

**Figure 2:**
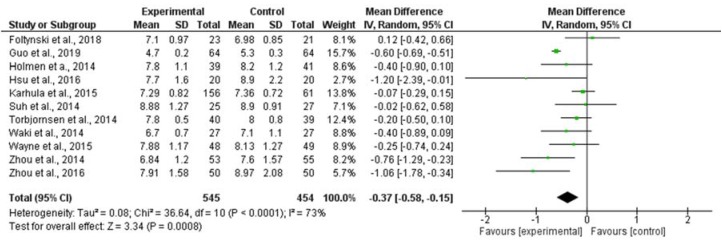
Comparison between intervention and control forestplot.

The meta-analytic estimation of the HbA1c % mean differences between intervention and control groups was statistically significant in favour of the intervention group with a mean difference of –0.37% (–0.58, –0.15. 95% confidence interval), as shown in Figure 2.

## 4. Discussion

In the systematic review described, data were obtained for people with DM1, DM2, and gestational diabetes. All of the studies included were clinical trials, presenting high methodological quality and low levels of bias, thus ensuring good internal validity [11]. The number of studies is high in comparison with other reviews with meta-analysis of diabetes, however, the number could be higher, but the difficulty of getting study populations for clinical trials, and the economic costs of a follow-up and the technology devices may have influenced the number of studies. The study aim was to determine if the use of smartphone applications improves the level of glycosylated hemoglobin (HbA1c), which has been confirmed by the meta-analysis with a lower HbA1c level mean difference between the intervention and control groups of 0.37 (–0.58, –0.15. 95% confidence interval), which is in concordance with older studies related to the topic [31]. The impact of these applications on glucose control and HbA1c-level reduction may be higher in the coming years, because the technology for self-monitoring blood glucose and continuous glucose monitoring is growing and improving in its automatization [9]. 

However, not only the development of clinical trials is important for the improvement of mHealth apps for diabetes. The usefulness of diabetes apps should be tested in real clinical care and real-life conditions to extract valued data, such as the mySurg app is doing by reaching more than 1.5 million people with diabetes and showing positive results in the glucose control of people with DM1 [9].

Regarding patients diagnosed with DM1, several studies have reported that smartphone apps and feedback provide vital assistance in keeping blood sugar levels within the appropriate range. However, insulin recommendations are only beneficial when there is an up-to-date, accurate record of health data. Accordingly, for satisfactory results to be obtained, the patients must make correct use of the technology. These apps can also notify the user of inadvertent hypoglycemia, so that appropriate steps can be taken in good time. On the other hand, to provide the app with appropriate data and to properly interpret the information received, the patient must accurately determine the carbohydrate content of the food consumed and be able to manage the insulin treatment competently. Nevertheless, in the medium term, the appropriate use of an app such as those described results in better stabilization of blood sugar levels and reduced HbA1c [32,33].

Various studies have considered the use of the rapid insulin bolus calculator for patients with DM1, concluding that it contributes to reducing HbA1c levels by an average of 0.91% for poorly controlled patients. In the case of well-controlled patients, although HbA1c levels do not change significantly, there is a decreased risk of post-prandial hypoglycemia, and treatment satisfaction is increased [34,35]. Other systems, such as the synchronisation of the blood glucose sensor and the continuous closed-loop insulin infusion pump, also prevent the risk of hypoglycemia and improve patients’ quality of life. The latter system autonomously regulates the infusion of basal insulin according to the blood sugar level, although the intake must be determined using the bolus calculator. Perceived satisfaction with this system is high [36].

With respect to the artificial pancreas, to date, insufficient studies have been conducted for reliable conclusions to be drawn concerning any association between increased heart rate and the risk of hypoglycemia during and after physical exercise. However, one study has observed that an adjustment of the baseline insulin dose, decreasing it before exercise, reduces the risk of hypoglycemia during the three hours after performing such exercise [37].

Patients with DM2 can use smartphone apps for assistance with changes in lifestyles. In this respect, several authors have analyzed the follow-up of patients using phone calls or text messages. This feedback enables the professional to advise on the measures needed to correct blood sugar levels or to maintain them within the appropriate range. The advice given may concern diet, medication, and health-giving practices such as increased physical activity [38,39,40].

It has also been reported that appropriate medication, dietary changes, and/or increased physical exercise can significantly decrease HbA1c levels, directly benefiting patients with diabetes. One way to reach a greater number of patients is to make greater use of technological resources, but some patients have highlighted certain difficulties encountered, for example, in recording the necessary health data into a smartphone app. Such problems can lead to the patient abandoning the app and failing to perform the activities proposed [41].

For patients with DM2 who require complementary rapid insulin treatment, there exist smartphone apps that provide personalized recommendations, such as insulin bolus calculation. The data needed for this are obtained progressively by dietary control and by counting carbohydrate intake [42].

Other studies have described more complex interventions to improve HbA1c, such as participation in a cookery workshop. The idea underlying this type of initiative is to devise a meal plan that provides a diet suited to people with diabetes and which they can prepare themselves. Although no significant results have yet been obtained in this field, this line of research is of considerable interest, prioritizing self-care and enhancing the patient’s quality of life. Moreover, smartphone apps can be used to design a specific plan for controlling the blood sugar level [43,44]. Finally, all this helps prevent and decrease the prevalence of diabetes [45]. 

This study has some limitations. First, not all people with diabetes know how to use smartphone apps, thus, the results must be taken in account for people with diabetes who know how to use the apps. Another diabetes outcomes, such as continuous and flash glucose measurements and their impact on the field of apps, have not been covered in this study. It is important to indicate that flash glucose monitoring systems, using devices that show information for diabetes management such as the levels of blood glucose (some of them in real time), such as Abbott FreeStyle Optium Neodexcom G5 Mobile System and Ipro2 [46,47,48,49], are showing good results in diabetes managementand patient satisfaction. Some of these devices have an application for mobile phones. However, the studies that we have found have not used the app or analyzed the impact of using that app on HbA1c. Thus, it should be investigated in the future. Also, the meta-analysis results are based on people with diabetes 1 and diabetes 2 so, although the complications related to glucose levels and protective health habits are mostly the same, the apps may be more effective for one population than the other. Finally, due to the moderate level of heterogeneity of the meta-analysis [50], which means that there are differences between the results of each clinical trial included, the results must be taken into account with caution. In the future, research should also analyze and compare if the effect of an intervention is clearly due to the app’s influence or due to the telemedicine elements (calls and text messages) that some apps use. 

## 5. Conclusions

Smartphone apps can help people with diabetes to improve and reduce their levels of HbA1c, but the clinical impact on the % of HbA1c is low. The applications included in the literature use different mechanisms such as algorithms to alert users about abnormal glucose levels, education and information about diabetes, counseling or healthy lifestyle advice to promote the self-management of diabetes and glucose control, and telemedicine elements. The interpretation of the results must take into account the knowledge of the target population about the use of smartphones and apps.

## Figures and Tables

**Table 1:** Studies obtained from the research.

Author, Country (Year)	Design	Sample	Intervention	Main Results	Level of Evidence/Degree of Education
Anzaldo-Campos et al., Mexico (2016)	Randomized Clinical Trial	Diabetes mellitus 2 patients. Control group (*n* = 100) Intervention group (*n* = 102).	Project Dulce technology-enhaced intervention (10 months). Care management by multidisciplinary team, and peer-led group education component. Glucose levels were send by the cell phone and patients received text messages, short educational videos, brochures, and took interactive surveys.	The HbA1c levels statiscally decreased 3.02% after 10 months while in the control group the decrease was 1.30%. The difference between both groups was statistically significant.	1a/A
Arora et al., USA (2014)	Randomized Clinical Trial	Diabetes mellitus 1 and 2 patients. Control group (*n* = 64) Intervention group (*n* = 64).	TExT-MED (6 months). Daily text messages delivered to patients’ mobile phones. The messages enhanced patient motivation, self-efficacy, and ability to perform diabetes self-care behaviors, including information about diabetes, medication reminders, healthy-living challenges, and questions. Two messages were send twice a day (9 AM and 5 PM).	HbA1c decreased by 1.05% in the intervention group and 0.6% in the control group after 6 months. The mean difference was not statistically significant between groups.	1a/A
Drion et al., Netherlands (2015).	Randomized Clinical Trial	Diabetes mellitus 1 patients. Control group (*n* = 32) Intervention group (*n* = 31).	Diabetes Under Control app (3 months). The app let patients introduce diabetes-related self-care data (blood glucose, carbohydrate intake, medication, physical exercise, and notes).	No significant differences were found between groups in the HbA1c levels after the intervention.	1a/A
Fang, China (2018)	Randomized clinical trial	Diabetes mellitus 2 patients. Control group (*n* = 62) Intervention group (*n* = 67).	The intervention group received microletters and short messages with diabetes related information while the control group received short calls.	After 1 year the intervention group level of HbA1c decreased from 6.96 (± 1.30) in pre-intervention to 6.55 ± 1.06 in post-intervention (*p* < 0.05). In the control group the HbA1c level increased (*p* > 0.05). The difference in HbA1c levels between groups in pre- and post-intervention were statistically significant.	1a/A
Foltynski, Polonia (2018)	Randomized clinical trial (crossed)	Diabetes mellitus 1 patients. Control group (*n* = 27) Intervention group (*n* = 27).	During Period 1 (4 d) a group received support from the VoiceDiab system in insulin bolus calculations (Treatment A) or performed manual bolus calculations (Treatment B). At 14 d, the patients move to Period 2 (4 d) and received the opposite treatment plan to that received in Period 1.	No differences in the HbA1c levels were found between groups.	1a/A
Guo et al., China (2019)	Randomized clinical trial	Pregnant women with gestiational diabetes mellitus. Control group (*n* = 60) Intervention group, mHealth group (*n* = 64).	The intervention group had the application “Dnurse App” to monitor fasting and post-prandial glucose. It also included an online instruction done by a nurse every day for 2 h to answer questions about diet, exercise, blood glucose, and other relevant topics. They were notified if they uploaded an abnormal blood glucose result.	The HbA1c mean was lower in the intervention group (4.07) than in the control group (5.3) with *p* < 0.001.	1a/A
Holmen et al., Norway (2014)	Three arm randomized controlled clinical trial	Diabetes mellitus 2 patients. Control group (*n* = 50) Intervention group 1: Few Touch App group, (*n* = 51).Intervention group 2: Few Touch with Health Counseling group (*n* = 50).	The Few Touch App provided the user a diabetes diary app designed to increase self-managament through awareness, overview of relevant factors, and motivational feedback. It also included registration of food and physical-activity habits. The other group added Health Counseling to the app for the first 4 months.	After one year, the level of HbA1c decreases in all groups. However, the change in the level of HbA1c did not differ significantly between groups.	1a/A
Hsu et al., USA (2016)	Randomized clinical trial	Diabetes mellitus 2 patients. Control group (*n* = 20) Intervention group (*n* = 20).	A cloud-based diabetes management program was used for 14 w by people with diabetes through an app. It included self-tracking informing about medication, medication adherence, and blood glucose and it emphasized other factors like diet and exercise. The app also included weekly charts with healthcare professionals (virtual visits with audio, video, and shared screen control).	Both groups decreased their levels of HbA1c after the intervention. The decrease in the intervention groups was higher than in the control group (3.2 vs. 2.0) with *p* < 0.05.	1a/A
Karhula et al., Finland (2015)	Randomized clinical trial	Diabetes mellitus 2 patients. Control group (*n* = 70) Intervention group (*n* = 180).	Mobile personal health record app. The patients sent health information through the app once a week.	After 1 year, no significant statistical differences were found in HbA1c levels between groups or in pre- andpost-intervention levels in the intervention group.	1a/A
Kim et al., Korea (2019)	Randomized clinical trial	Diabetes mellitus 2 patients. mDiabetes group (*n* = 90) pLog-book group (*n* = 82).	The mDiabetes was used for 24 w (application that used an algorithm to send inmediate feedback messages according to the glycemic control).	The reduction of HbA1c was higher in the experimental group -0.40 compared to the control group -0.06. The mean difference between groups was 0.35% (*p* < 0.05).	1a/A
Klee et al., Switzerland (2018)	Randomized clinical trial	Diabetes mellitus 1 patients (children and adolescents). Experimental group used Webdia app (*n* = 20) Control group (*n* = 13).	Webdia app to help in calculating the insulin dose and information about meals. Patients used the app for 3 months.	After 3 months, when taking into account patients with HbA1c higher than 8% the reduction in the intervention group was 0.33 while the control group had increased their HbA1c level (0.21) with *p* < 0.05.	1a/A
Skrovseth et al., Norway (2015)	Randomized clinical trial	Diabetes mellitus 1 patients. Control group (*n* = 15) Intervention group (*n* = 15).	Diabetes Diary App (18 w). The app included a daily and weekly blood glucose graphic, blood glucose trends, situations matching when injecting insulin, physical activity, and carbohydrate registration.	Both groups decreased their levels of HbA1c, but the differences in the mean values of HbA1c were not statistically significant.	1a/A
Suh, Korea (2014)	Randomized clinical trial	Diabetes mellitus 1 patients. Control group (*n* = 27) Intervention group (*n* = 25).	The intervention group received individual feedback on their results. They sent the results of their glucometer to a website. Then they received support by a mentor on insulin dosing, physical activity, and food intake within 48 h of transmission. Text messages were sent to notify the assigned mentors when their trainees uploaded their data. The control group did not receive any comments but were able to review and interpret their own website data as often as necessary.	After 12 w the control group HbAc1 level decreased from 9.52 ± 1.01 to 8.9 ± 0.91. After 12 w the intervention group HbAc1 decreased from 9.39 ± 1.21 to 8.88 ± 1.27. Without statistical significance the subjects who improved their HbA1c levels more than 1% were 68.8% of patients in the intervention group vs. 41.7% in the control group.	1a/A
Torbjornsen et al., Norway (2014)	Randomized clinical trial	Diabetes mellitus 2 patients. Control group (*n* = 44) Intervention group (*n* = 42).	Few Touch Application (4 months). It is a diary for type 2 diabetes included in the smartphone. The app registered blood glucose, food habits, physical activity, personal goal-setting system and general diabetes information.	The mean level of HbA1c decreased in all the intervention group and control group (0.23 vs. 0.39), but the mean difference was not statistically significant.	1a/A
Waki, Japan (2014)	Randomized clinical trial	Diabetes mellitus 2 patients. Control group (*n* = 27) Intervention group (*n* = 27).	DialBetics consisted of 4 modules:(1) data transmission module; (2) evaluation module; (3) communication module; (4) dietary evaluation module. A 3-month study was designed to assess safety and usability.	After 3 months, the DialBetics Group HbA1c decreased 0.4%, while in the Non-DiaBeltics Group it increased 0.1%. A statistically signifcant difference in HbA1c was found between groups after the intervention.	1a/A
Wayne et al., Canada (2015)	Randomized clinical trial	Diabetes mellitus 2 patients. Control group (*n* = 49) Intervention group (*n* = 48).	For 6 months patients recorded their information about blood glucose, physical activity, food intake, and mood in the app. Patients could comunnicate with a health coach through their phone (messaging, calls, and person meetings). The health coach could see the patients information.	After 6 months, both groups decreased their levels of HbA1c, but no significant differences were found between intervention and control group reductions (0.84% vs. 0.81%)	1a/A
Zhou, China (2014)	Randomized clinical trial	Diabetes mellitus 2 patients. Control group (*n* = 55) Intervention group (*n* = 53).	Telemedicine group and a traditional group of face-to-face visits as a control	After 3 months, the telemedicine intervention HbA1c levels decreased in the control group from 8.22 ± 1.58 to 7.6 ± 1.57 and in the intervention group from 8.44 ± 1.58 to 6.84 ± 1.2. There was no significant difference between groups.	1a/A
Zhou et al., China (2016)	Randomized clinical trial	Diabetes mellitus 1 and 2 patients. Control group (*n* = 50) Intervention group (*n* = 50).	Welltang app for 3 months. This was a diabetes management application and could be used by patients and clinicians. It had three main aims: knowledge, self-management, and communication between patients and clinicians.	After 3 months follow-up, the HbA1c mean was 7.91(±1.58) for the intervention group and 8.97(± 2.08) for the control group. The differences between groups were significant (*p* < 0.01).	1a/A

HbAc1 = glycosylated hemoglobin.

## Supplementary Materials

The following are available online at https://www.mdpi.com/2077-0383/9/3/693/s1, File S1: PUBMED REFERENCED.
