# Supplementary material for: Smartphones and Apps to Control Glycosylated Hemoglobin (HbA1c) Level in Diabetes: A Systematic Review and Meta-Analysis
Source: J Clin Med. 2020 Mar 4;9(3):693. doi: 10.3390/jcm9030693 (PMC7141208; doi:10.3390/jcm9030693)
Supplement: Supplementary file 1 [file jcm-09-00693-s001.pdf]

PUBMED REFERENCED

STUDY PROTOCOL
1. BMC Public Health. 2019 Oct 15;19(1):1287. doi: 10.1186/s12889-019-7691-3.
Smart Phone APP to Restore Optimal Weight (SPAROW): protocol for a randomised controlled trial for women with recent gestational diabetes.
Lim K(1), Chi C(1), Chan SY(1), Lim SL(2), Ang SM(2), Yoong JS(3), Tsai C(4), Wong SR(4), Yew TW(5), Tai ES(5), Yong EL(6).

STUDY PROTOCOL
2. Trials. 2019 Aug 22;20(1):521. doi: 10.1186/s13063-019-3623-x.

Study protocol for a randomized controlled trial to test for preventive effects of diabetic foot ulceration by telemedicine that includes sensor-equipped insoles combined with photo documentation.
Ming A(1), Walter I(1), Alhajjar A(1), Leuckert M(1), Mertens PR(2).

NON DIABETICS SAMPLE
3. Diabetes Technol Ther. 2019 Sep;21(9):507-513. doi: 10.1089/dia.2019.0134. Epub 2019 Jun 11.
Engagement and Weight Loss: Results from the Mobile Health and Diabetes Trial.
Muralidharan S(1)(2), Ranjani H(2), Mohan Anjana R(2), Jena S(3), Tandon N(4), Gupta Y(4), Ambekar S(4), Koppikar V(3), Jagannathan N(2), Allender S(1), Mohan V(2).

PATIENTS WITH STROKE
4. Stroke. 2019 Jul;50(7):1819-1824. doi: 10.1161/STROKEAHA.118.024355. Epub 2019 Jun 6.
Farmalarm. Requena M(1)(2), Montiel E(1), Baladas M(1), Muchada M(1), Boned S(1)(2), Lopez R(3), Rodriguez-Villatoro N(1)(2), Juega J(1)(2), Garcia-Tornel √Å(1),Rodriguez-Luna D(1)(2), Pagola J(1)(2), Rubiera M(1)(2), Molina CA(1)(2), Ribo M(1)(2).

SYSTEMATIC REVIEW, USED FOR INVERSE SEARCH
5. Comput Inform Nurs. 2019 Jul;37(7):340-348. doi: 10.1097/CIN.0000000000000526. Structure and Characteristics of Diabetes Self-Management Applications: A Systematic Review of the Literature. Angelini S(1), Alicastro GM, Dionisi S, Di Muzio M.

SYSTEMATIC REVIEW, USED FOR INVERSE SEARCH
6. JMIR Mhealth Uhealth. 2019 Jan 15;7(1): e12297. doi: 10.2196/12297.
The Efficacy of Mobile Phone Apps for Lifestyle Modification in Diabetes: Systematic Review and Meta-Analysis.
Wu X(1), Guo X(1), Zhang Z(2).

NO CLINICAL TRIAL
7. J Clin Med. 2019 Jan 17;8(1). pii: E109. doi: 10.3390/jcm8010109.
The Role of Continuous Glucose Monitoring, Diabetes Smartphone Applications, and Self-Care Behavior in Glycemic Control: Results of a Multi-National Online Survey.

Kebede MM(1)(2)(3), Schuett C(4), Pischke CR(5)(6).

NO CLINICAL TRIAL
8. Clin Cardiol. 2019 Feb;42(2):217-221. doi: 10.1002/clc.23124. Epub 2019 Jan 17.
SMARTWOMAN,Ñ¢: Feasibility assessment of a smartphone app to control cardiovascular risk factors in vulnerable diabetic women.
Wenger NK(1), Williams OO(2), Parashar S(3).

INCLUDED
9. Diabetes Technol Ther. 2018 Dec;20(12): 797-805. doi: 10.1089/dia.2018.0255. Epub 2018 Nov 7. An Intervention by a Patient-Designed Do-It-Yourself Mobile Device App Reduces HbA1c in Children and Adolescents with Type 1 Diabetes: A Randomized Double-Crossover Study.
Klee P(1)(2), Bussien C(3), Castellsague M(4), Combescure C(5), Dirlewanger M(1)(2), Girardin C(1)(2), Mando JL(6), Perrenoud L(4), Salomon C(7), Schneider F(8), Schwitzgebel VM(1)(2).

STUDY PROTOCOL
10. BMC Endocr Disord. 2018 Oct 22;18(1):74. doi: 10.1186/s12902-018-0304-9.
Effectiveness of diabetes self-management education via a smartphone application in insulin treated type 2 diabetes patients - design of a randomised controlled trial ('TRIGGER study').
Boels AM(1), Rutten G(2), Zuithoff N(2), de Wit A(2)(3), Vos R(2)(4).

STUDY PROTCOL AND NO DIABETIC PATIENTS
11. Ther Adv Endocrinol Metab. 2018 Jun;9(6):167-176. doi: 10.1177/2042018818770938.
Epub 2018 Apr 27. Efficacy of gamification-based smartphone application for weight loss in overweight and obese adolescents: study protocol for a phase II randomized controlled trial.
Timpel P(1), Cesena FHY(2), da Silva Costa C(3), Soldatelli MD(4), Gois E Jr(5), Castrillon E(6), Diaz LJJ(7), Repetto GM(8), Hagos F(9), Castillo Yermenos RE(10), Pacheco-Barrios K(11), Musallam W(12), Braid Z(13), Khidir N(14), Romo Guardado M(15), Roepke RML(16).

NO DIABETICS PATIENTS
12. J Am Board Fam Med. 2018 May-Jun;31(3):364-374. doi: 10.3122/jabfm.2018.03.170280. A Latino Patient-Centered, Evidence-Based Approach to Diabetes Prevention. Rosas LG(1), Lv N(2), Lewis MA(2), Venditti EM(2), Zavella P(2), Luna V(2), Ma J(2).

NO DIABETICS PATIENTS
13. Obstet Gynecol. 2018 May;131(5):818-826. doi: 10.1097/AOG.0000000000002582. Pregnancy Exercise and Nutrition with Smartphone Application Support: A Randomized Controlled Trial. Kennelly MA(1), Ainscough K, Lindsay KL, O ºSullivan E, Gibney ER, McCarthy M,Segurado R, DeVito G, Maguire O, Smith T, Hatunic M, McAuliffe FM.

NOT RELATED WITH STUDY TOPIC
14. Eye (Lond). 2018 Jun;32(6):1138-1144. doi: 10.1038/s41433-018-0064-9. Epub 2018 Mar 9. Automated diabetic retinopathy detection in smartphone-based fundus photography using artificial intelligence. Rajalakshmi R(1), Subashini R(2), Anjana RM(2), Mohan V(2).

NO DIABETICS PATIENTS
15. Transl Behav Med. 2018 Sep 8;8(5):714-723. doi: 10.1093/tbm/ibx039.
A weight loss intervention using a commercial mobile application in Latino Americans-Adelgaza Trial. Fukuoka Y(1), Vittinghoff E(2), Hooper J(3).

COMMENTARY, NO STUDY.
16. Diabetes Metab Res Rev. 2018 Feb;34(2). doi: 10.1002/dmrr.2985. Diabetes on demand and novel technologies. Maurizi AR(1), Piemonte V(2), Pozzilli P(1)(3).

STUDY PROTOCOL
17. BMC Public Health. 2018 Jan 10;18(1):119. doi: 10.1186/s12889-018-5026-4. App-technology to increase physical activity among patients with diabetes type 2 - the DiaCert-study, a randomized controlled trial. Bonn SE(1), Alexandrou C(2)(3), Hj√∂rleifsdottir Steiner K(4), Wiklander K(2), √ñstenson CG(5), L√∂f M(3), Trolle Lagerros Y(2)(6).

INCLUDED
18. J Diabetes. 2018 Jul;10(7):600-608. doi: 10.1111/1753-0407.12641. Epub 2018 Feb
9. Efficacy of automatic bolus calculator with automatic speech recognition in
patients with type 1 diabetes: A randomized cross-over trial. Foltynski P(1), Ladyzynski P(1), Pankowska E(2), Mazurczak K(2).

NO INFORMATION ABOUT HbA1c
19. J Diabetes Sci Technol. 2018 Nov;12(6):1192-1199. doi: 10.1177/1932296817749859.
Epub 2018 Jan 1. Use of a Smartphone Application to Reduce Hypoglycemia in Type 1 Diabetes: A Pilot Study. Feuerstein-Simon C(1), Bzdick S(1), Padmanabhuni A(1), Bains P(1), Roe C(1), Weinstock RS(1).

NOT RELATED WITH THE STUDY TOPIC
20. Diabet Med. 2018 Apr;35(4):436-449. doi: 10.1111/dme.13567. Epub 2018 Feb 15. Psychosocial impacts of hybrid closed-loop systems in the management of diabetes:
a review. Farrington C(1).

STUDY PROTOCOL
21. BMJ Open. 2017 Oct 8;7(10):e018282. doi: 10.1136/bmjopen-2017-018282.
Sensing interstitial glucose to nudge active lifestyles (SIGNAL): feasibility of combining novel self-monitoring technologies for persuasive behaviour change. Whelan ME(1)(2), Kingsnorth AP(1)(2), Orme MW(3), Sherar LB(1)(2)(4), Esliger DW(1)(2)(4).

NO PATIENTS WITH DIABETES AND REVIEW
22. Curr Diab Rep. 2017 Sep 23;17(11):107. doi: 10.1007/s11892-017-0948-2. A Review of Technology-Assisted Interventions for Diabetes Prevention.
Grock S(1), Ku JH(2), Kim J(2), Moin T(3).

STUDY PROTOCOL
23. J Adv Nurs. 2018 Jan;74(1):190-200. doi: 10.1111/jan.13394. Epub 2017 Aug 17. A randomized controlled trial on a nurse-led smartphone-based self-management
programme for people with poorly controlled type 2 diabetes: A study protocol. Wang W(1), Seah B(1), Jiang Y(1), Lopez V(1), Tan C(2), Lim ST(3), Ren H(4), Khoo

YH(3).

STUDY PROTOCOL

24. BMC Med Inform Decis Mak. 2017 Jul 18;17(1):109. doi: 10.1186/s12911-017-0507-4. An information and communication technology-based centralized clinical trial to determine the efficacy and safety of insulin dose adjustment education based on a smartphone personal health record application: a randomized controlled trial. Kim G(1), Bae JC(2), Yi BK(3), Hur KY(1), Chang DK(3)(4), Lee MK(1), Kim JH(5)(6), Jin SM(7)(8).

INCLUDED

25. J Clin Nurs. 2018 Feb;27(3-4):612-620. doi: 10.1111/jocn.13962. Epub 2018 Jan 8. Electronic messaging intervention for management of cardiovascular risk factors in type 2 diabetes mellitus: A randomised controlled trial. Fang R(1), Deng X(1).

NOT RELATED TO THE TOPIC AND IT IS A COMMENTARY

26. Prim Care Diabetes. 2017 Aug;11(4):397-399. doi: 10.1016/j.pcd.2017.03.008. Epub 2017 Apr 28. Pokemon GO: An innovative smartphone gaming application with health benefits. Kamboj AK(1), Krishna SG(2).

ARTICLE IN SWEDISH AND NOT RELATED TO THE TOPIC

27. Lakartidningen. 2017 Apr 25;114. pii: EFF9. [Energy restriction and adherence required for weight loss without surgery]. [Article in Swedish] Larsson I(1), Sandqvist M(2), Werling M(3), Wiklund M(4), Bergh C(5), Eliasson B(6).

COMMENTARY, NO DATA

28. Diabetes Technol Ther. 2016 Nov;18(11):725-726. Epub 2016 Oct 18. Pok√©mon Go, Obesity and Diabetes: A Perspective from India. Ghosh A(1), Misra A(1)(2)(3).

PATIENTS WITHOUTH DIABETES, IT IS A CONGRESS POSTER

29. Stud Health Technol Inform. 2016;225:1034-5. Preliminary Findings of the Delivery of the National Diabetes Prevention Program via a Mobile Application. Tiase VL(1), Licata M(2), Fleck EM(2).

REVIEW WITH STUDIES OLDER THAN 5 YEARS.

30. Int J Clin Pract. 2016 Jun;70(6):434-49. doi: 10.1111/ijcp.12816. Innovative health informatics as an effective modern strategy in diabetes management: a critical review. David SK(1), Rafiullah MR(2).

NO CLINICAL TRIAL

31. Diabetes Care. 2016 Jul;39(7):1143-50. doi: 10.2337/dc15-2468. Epub 2016 Apr 13. Multinational Home Use of Closed-Loop Control Is Safe and Effective. Anderson SM(1), Raghinaru D(2), Pinsker JE(3), Boscari F(4), Renard E(5), Buckingham BA(6), Nimri R(7), Doyle FJ 3rd(8), Brown SA(1), Keith-Hynes P(9).

NO CLINICAL TRIAL, NO DATA ABOUT HbA1c

32. J Diabetes Sci Technol. 2017 Jan;11(1):182-183. doi: 10.1177/1932296816650901. Epub 2016 Jul 9. Use of a Novel Smartphone-Based Diabetes Management System Improved Feelings of

Confidence and Safety and Reduced Hypoglycemia Fear Among Parents/Caregivers of Children/Adolescents with Type 1 Diabetes. Prakasam G(1), Rees C(2), Lyden M(3), Parkin CG(4).

STUDY PROTOCOL
33. Trials. 2016 Apr 27;17(1):215. doi: 10.1186/s13063-016-1345-x. A complex behavioural change intervention to reduce the risk of diabetes and prediabetes in the pre-conception period in Malaysia: study protocol for a randomised controlled trial. Skau JK(1), Nordin AB(2), Cheah JC(3), Ali R(2), Zainal R(4), Aris T(5), Ali
ZM(6), Matzen P(7), Biesma R(7)(8), Aagaard-Hansen J(9)(10), Hanson MA(7), Norris
SA(9).

NO CLINICAL TRIAL
34. Diabetes Self Manag. 2016 Mar-Apr;33(2):28-31. WEARABLES, IMPLANTS AND APPS, Oh My! SMART TECHNOLOGY FOR DIABETES SELF-CARE. Davies N.

INCLUDED
35. JMIR Diabetes. 2016 Apr 6;1(1): e1. doi: 10.2196/diabetes.4506.
Data Mining of a Remote Behavioral Tracking System for Type 2 Diabetes Patients: A Prospective Cohort Study. Wayne N(#)(1), Cercone N(#)(2), Li J(#)(2), Zohar A(#)(1), Katz J(#)(3), Brown P(#)(4)(5), Ritvo P(#)(1).

REVIEW WITHOUTH RTC FOR OUR REVIEW
36. Prog Cardiovasc Dis. 2016 May-Jun;58(6):630-8. doi: 10.1016/j.pcad.2016.03.001.
Epub 2016 Mar 6. Adapting Technological Interventions to Meet the Needs of Priority Populations. Linke SE(1), Larsen BA(2), Marquez B(2), Mendoza-Vasconez A(2), Marcus BH(2).

LETTER TO THE EDITOR
37. Can J Diabetes. 2016 Apr;40(2):108. doi: 10.1016/j.jcjd.2015.08.008. Epub 2016 Jan 6. Smartphone for Managing Diabetes. Joob B(1), Wiwanitkit V(1). Author information:
(1) Sanitation 1 Medical Academic Center, Bangkok, Thailand.

STUDY PROTOCOL
38. Contemp Clin Trials. 2016 Jan;46:92-99. doi: 10.1016/j.cct.2015.11.018. Epub 2015 Nov 25. Pregnancy, exercise and nutrition research study with smart phone app support(Pears): Study protocol of a randomized controlled trial. Kennelly MA(1), Ainscough K(2), Lindsay K(2), Gibney E(3), Mc Carthy M(4),
McAuliffe FM(2).

REVIEW USED FOR INVERSE SEARCH
39. Circulation. 2015 Nov 24;132(21):2012-27. doi: 10.1161/CIRCULATIONAHA.114.008723. Mobile Health Devices as Tools for Worldwide Cardiovascular Risk Reduction and
Disease Management. Piette JD(1), List J(2), Rana GK(2), Townsend W(2), Striplin D(2), Heisler M(2).

NON DIABETIC PATIENTS
40. J Med Internet Res. 2015 Oct 23;17(10): e240. doi: 10.2196/jmir.4897. Diabetes Prevention and Weight Loss with a Fully Automated Behavioral

Intervention by Email, Web, and Mobile Phone: A Randomized Controlled Trial Among Persons with Prediabetes. Block G(1), Azar KM, Romanelli RJ, Block TJ, Hopkins D, Carpenter HA, Dolginsky
MS, Hudes ML, Palaniappan LP, Block CH.

NO SMARTPHONE USE
41. Lancet Diabetes Endocrinol. 2015 Dec;3(12):939-47. doi: 10.1016/S2213-8587(15)00335-6. Epub 2015 Sep 30. Two month evening and night closed-loop glucose control in patients with type 1 diabetes under free-living conditions: a randomised crossover trial. Kropff J(1), Del Favero S(2), Place J(3), Toffanin C(4), Visentin R(2), Monaro
M(2), Messori M(4), Di Palma F(4), Lanzola G(5), Farret A(3), Boscari F(6),

NOT RELATED WITH STUDY TOPIC
42. PLoS One. 2015 Sep 24;10(9):e0138285. doi: 10.1371/journal.pone.0138285.
eCollection 2015. Validation of Smartphone Based Retinal Photography for Diabetic Retinopathy Screening. Rajalakshmi R(1), Arulmalar S(1), Usha M(1), Prathiba V(1), Kareemuddin KS(1), Anjana RM(1), Mohan V(1).

REVIEW USED FOR INVERSE SEARCH
43. Circulation. 2015 Sep 22;132(12):1157-213. doi: 10.1161/CIR.0000000000000232. Epub 2015 Aug 13. Current Science on Consumer Use of Mobile Health for Cardiovascular Disease
Prevention: A Scientific Statement from the American Heart Association.
Burke LE, Ma J, Azar KM, Bennett GG, Peterson ED, Zheng Y, Riley W, Stephens J,

NOT RELATED WITH STUDY TOPIC
44. J Diabetes Sci Technol. 2015 Aug 6;9(6):1313-20. doi: 10.1177/1932296815588559.The Role of Mobile Applications in Improving Alcohol Health Literacy in Young Adults with Type 1 Diabetes: Help or Hindrance? Tamony P(1), Holt R(1), Barnard K(2).

NO RCT
45. J Telemed Telecare. 2016 Apr;22(3):172-8. doi: 10.1177/1357633X15595178. Epub 2015 Jul 21. Participant experiences in a smartphone-based health coaching intervention for
type 2 diabetes: A qualitative inquiry. Pludwinski S(1), Ahmad F(2), Wayne N(1), Ritvo P(3).

MIXED SAMPLE
46. Circulation. 2015 Sep 1;132(9):815-24. doi: 10.1161/CIRCULATIONAHA.115.015373.
Epub 2015 Jul 17. A Cluster-Randomized, Controlled Trial of a Simplified Multifaceted Management Program for Individuals at High Cardiovascular Risk (SimCard Trial) in Rural Tibet, China, and Haryana, India. Tian M(1), Ajay VS(1), Dunzhu D(1), Hameed SS(1), Li X(1), Liu Z(1), Li C(1),
Chen H(1), Cho K(1), Li R(1), Zhao X(1), Jindal D(1), Rawal I(1), Ali MK(1),
Peterson ED(1), Ji J(1), Amarchand R(1), Krishnan A(1), Tandon N(1), Xu

STUDY PROTOCOL
47. Can J Diabetes. 2015 Jun;39(3):200-3. doi: 10.1016/j.jcjd.2015.04.002. Epub 2015 Apr 30.A smartphone-based cloud computing tool for managing type 1 diabetes in Ontarians. Baskaran V(1), Prescod F(2), Dong L(3).

REVIEW USED FOR INVERSE SEARCH

48. World J Diabetes. 2014 Oct 15;5(5):689-96. doi: 10.4239/wjd.v5.i5.689. Advances in management of type 1 diabetes mellitus. Aathira R(1), Jain V(1).
